# Potential therapeutic application of mesenchymal stem cell-derived exosomes in SARS-CoV-2 pneumonia

**DOI:** 10.1186/s13287-020-01866-6

**Published:** 2020-08-14

**Authors:** Ali Akbari, Jafar Rezaie

**Affiliations:** Solid Tumor Research Center, Cellular and Molecular Medicine Research Institute, Urmia University of Medical Sciences, Shafa St, Ershad Blvd., P.O. Box: 1138, Urmia, 57147 Iran

**Keywords:** Virus pneumonia, Mesenchymal stem cells, Exosomes, SARS-CoV-2

## Abstract

**Background:**

The outbreak of a new virus known as severe acute respiratory syndrome coronavirus 2 (SARS-CoV-2) has now become the main health concern all over the world. Since effective antiviral treatments have not been developed until now, SARS-CoV-2 is severely affecting countries and territories around the world.

**Methods:**

At the present review, articles in PubMed were searched with the following terms: mesenchymal stem cells, exosomes, coronavirus, and SARS-CoV-2, either alone or in a combination form. The most relevant selected functions were mesenchymal stem cell-derived exosomes and SARS-CoV-2 virus infection.

**Results:**

SARS-CoV-2 could damage pulmonary cells and induce secretion of different types of inflammatory cytokines. In the following, these cytokines trigger inflammation that damages the lungs and results in lethal acute respiratory distress syndrome (ARDS). The main characteristic of ARDS is the onset of inflammation in pulmonary, hyaline formation, pulmonary fibrosis, and edema. Mesenchymal stem cell-derived exosomes (MSC-Exo) are believed to have anti-inflammatory effects and immune-modulating capacity as well as the ability to induce tissue regeneration, suggesting a significant therapeutic opportunity that could be used to SARS-CoV-2 pneumonia treatment. Besides, exosomes may serve as a biomarker, drug delivery system, and vaccine for the management of the patient with SARS-CoV-2.

**Conclusion:**

MSC-Exo may serve as a promising tool in the treatment of SARS-CoV-2 pneumonia. However, further work needs to be carried out to confirm the efficacy of exosomes in the treatment of SARS-CoV-2 pneumonia.

## Background

The exploration of severe acute respiratory syndrome coronavirus 2 (SARS-CoV-2) in the city of Wuhan in China is done in December 2019. SARS-CoV-2 can transmit human to human and spread rapidly to other parts of China and then to other countries. The number of individuals with SARS-CoV-2 has rapidly increased in a few weeks; as of 8 July 2020, more than 11 million cases have been reported across 213 countries and territories, resulting in more than 500,000 deaths, and more than 4 million people have recovered [1]. The World Health Organization (WHO) recognized it on January 12 and named it “new novel coronavirus 2019 (2019-nCoV)”; therefore, coronavirus 2019 (2019-nCoV) and COVID-19 virus are stated as follows: 2019-nCoV is a common name, and SARS-CoV-2 is a classification name for this new emerging virus. Because of the increasing afflicted people, concerns about reserve restrictions, and emerging understanding on how to best treat SARS-CoV-2, healthcare systems have established minimal therapeutic cares for both hospital admission and mechanical ventilation [2]. Currently, there is a significant lack of antiviral agents that could be used specifically for the treatment of SARS-CoV-2 infection. Although symptomatic and supportive cares are recommended for severely infected individuals, those with advancing age and co-morbidities such as diabetes and heart diseases remain to be at high risk for adverse outcomes. Therefore, it is urgent to find a safe and effective therapeutic approach to patients with severe SARS-CoV-2 virus characterized by a severe acute respiratory impairment [3]. It was demonstrated that mesenchymal stem cells (MSCs) and their derivatives such as exosomes (MSC-Exo) considerably improved lung inflammation and pathological damage resulting from different types of lung injuries [4, 5]. MSC-Exo have been shown to harbor different proteins and RNAs that have therapeutic effects such as regenerative, anti-inflammatory, pro-angiogenic, immunomodulatory, and anti-fibrotic properties on damaged tissues [6–8]. Based on these pieces of evidence, in this review, we aimed to discuss the possible therapeutic application of exosomes in SARS-CoV-2 virus infection.

## Coronaviruses

Coronaviruses are enclosed, sphere-shaped, or pleomorphic viruses, containing single-strand positive-sense RNA genome which is the longest among the RNA viruses [9]. They belong to a large family of virus which is the leading cause of common cold and severe infection such as Middle East respiratory syndrome (MERS) and severe acute respiratory syndrome (SARS) [10–12]. Before the recent pandemic, six types of coronavirus species are documented to induce human respiratory diseases [13]. There are four different coronaviruses types, namely NL63, human coronaviruses 229E, HKU1, and OC43, which classically infect only the upper respiratory system and cause fairly light symptoms [14], whereas three other coronaviruses like SARS-CoV, MERS-CoV, and more recently identified SARS-CoV-2 can infect the lower respiratory system and cause pneumonia, which can be lethal. SARS-CoV-2 is a member of the betacoronavirus genus. It genetically resembles, among human coronaviruses, SARS-CoV-2 with 79% similarity [15], while among wholly recognized coronavirus sequences, it has about 98% similarity to bat coronavirus RaTG13 [16] and also share high similarity to coronavirus sequences of pangolin [17].

## Pathogenesis of SARS-CoV-2

**Fig. 1:**
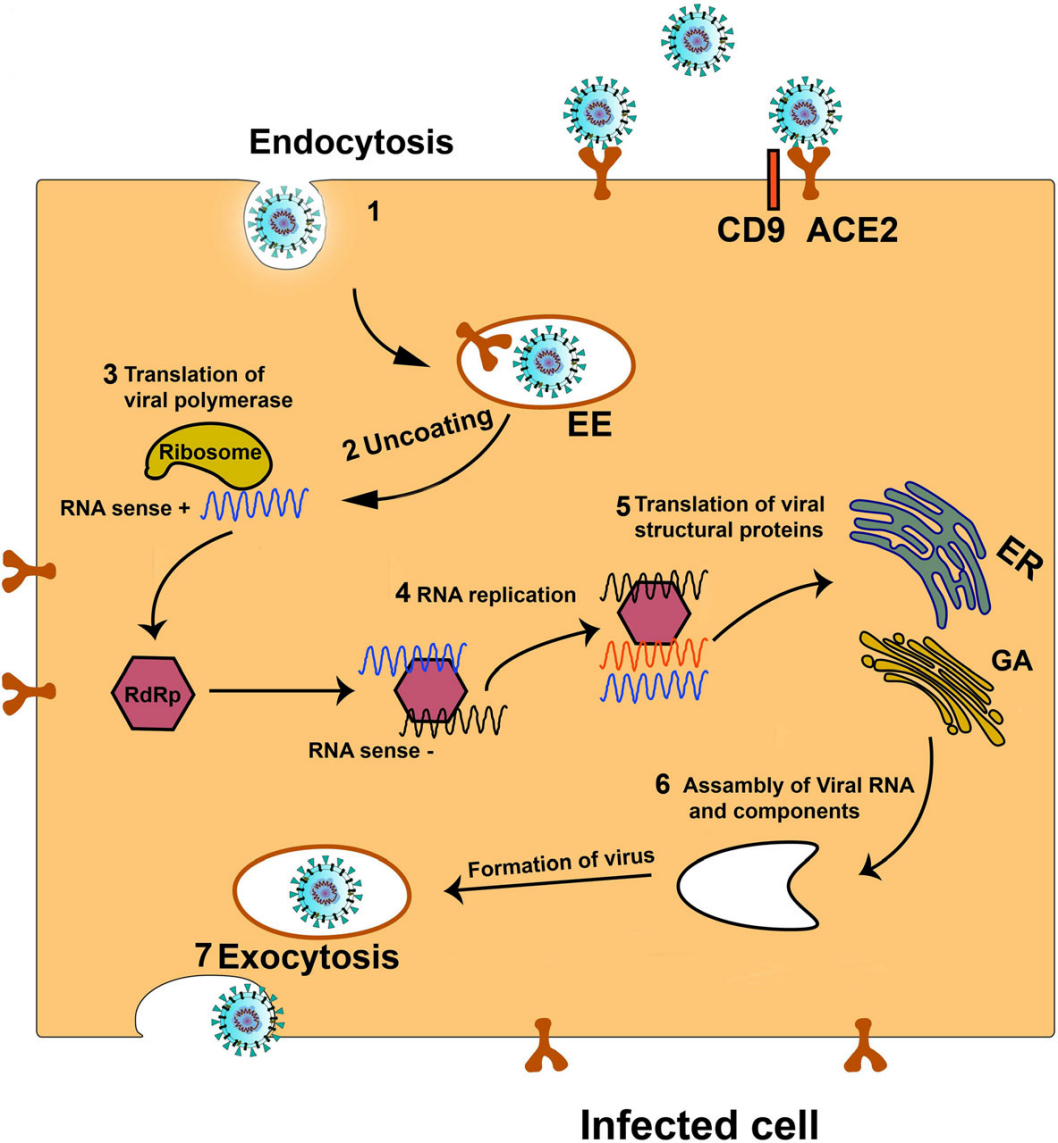
The SARS-CoV-2 entry and life cycle in human lung cells. The S proteins of SARS-CoV-2 bind to the angiotensin-converting enzyme 2 (ACE2) located on the cellular membrane and enters the target cells through an endosomal pathway (1). Then, viral components such as RNA are uncoated in the cytoplasm (2). In keeping, the genome encodes RNA-dependent RNA polymerase (RdRp) (3), which, in turn, participates to producing full-length (+) RNA and full length (−) RNA copies (4). During replication, genome RNA and RNAs which belong to viral structural components are produced. Then, RNAs are translated into viral components and processed by the endoplasmic reticulum (ER)–Golgi apparatus (GA) (5). Once SARS-CoV-2 structural proteins are formed, nucleocapsids initially are assembled in the cytoplasm, and then they are budding into the lumen of the ER and GA (6). Finally, mature viruses are secreted out of the infected cell via exocytosis (7). EE, early endosome

**Fig. 2:**
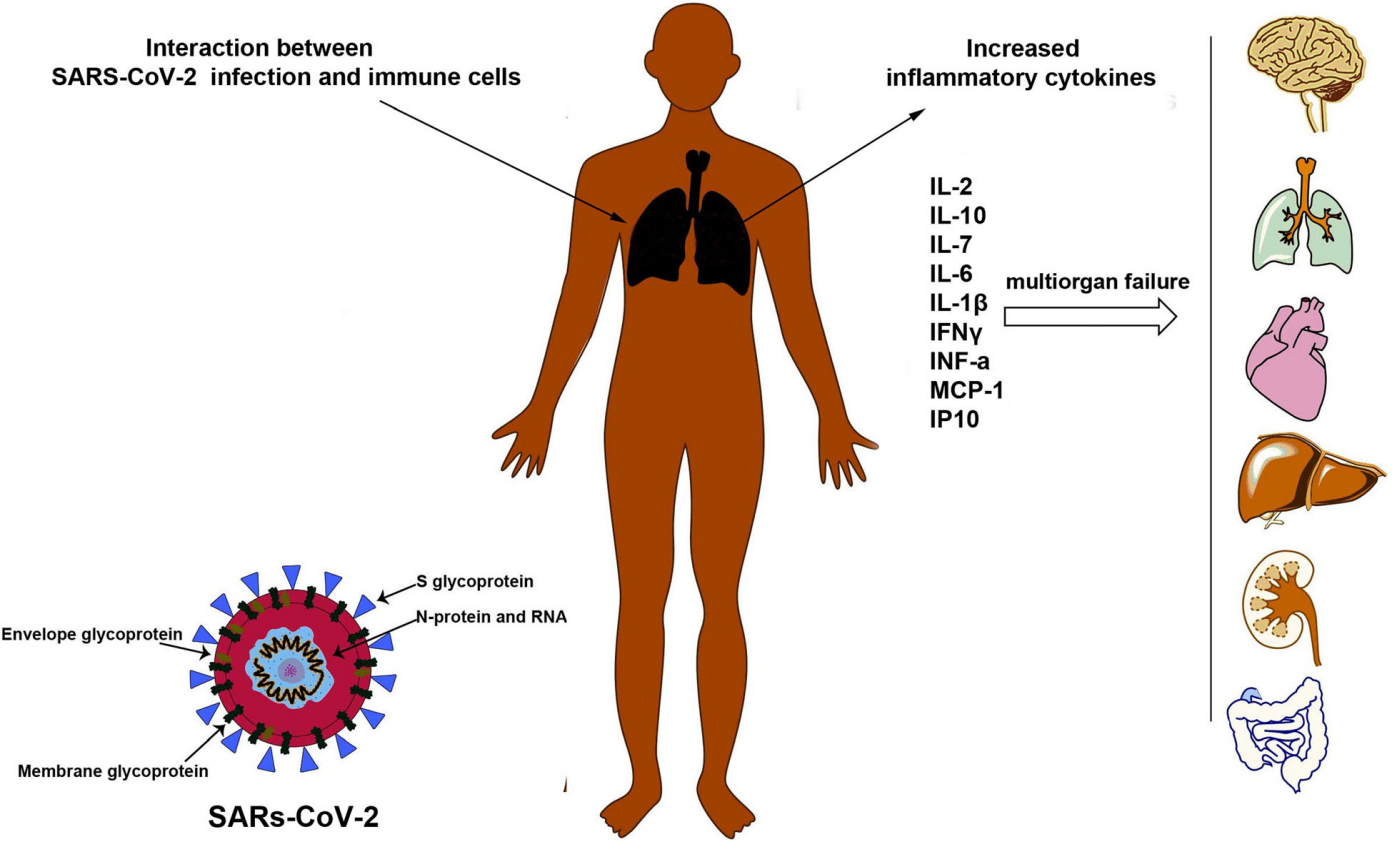
Pathogenesis of SARS-CoV-2 virus. During SARS-CoV-2 infection, immune cells produce several pro-inflammatory cytokines. Inflammatory cytokines trigger lethal acute respiratory distress syndrome (ARDS) and failure in multi-organs

The affliction of coronaviruses is mediated by a trimeric spike glycoprotein existing on the virion membrane. Similar to the envelope of HIV or hemagglutinin of influenza species, the spike proteins of coronavirus are one of class I fusion proteins [18]. SARS-CoV-2 enters into cells when the S protein binds to the ACE2 receptor located on the host cell membrane [19] (Fig. 1). After docking, the S protein conformation is changed, which facilitates virus entry into the endosomal pathway. Then, viral components such as RNA were uncoated inside the cell and translated into the viral components. Once the structural proteins of SARS-CoV-2 are formed, nucleocapsids initially are assembled in the cytoplasm and then inward bud into the lumen of the endoplasmic reticulum (ER)–Golgi transitional compartments. Then, SARS-CoV-2 structural proteins are released out of the infected cell via exocytosis [19] (Fig. 1). SARS-CoV-2 spreads mostly via respiratory droplets and via, likely, but unconfirmed, the orofecal route. During infection, the average latency is around 4–5 days earlier before the symptom begins [20–22]; however, 97.5% of the symptoms emerge within 11.5 days [22]. The results reported from hospitals indicate patients with SARS-CoV-2 basically show fever, dry cough, muscle and/or joint pain, difficulty in breathing, diarrhea, nausea, headache, and hemoptysis [23–26]. Between days 5 and 6 after the onset of symptoms, SARS-CoV-2 viral load rises to its peak—expressively earlier than that of the SARS-CoV one—so around 10 days later, the viral load peak reaches maximum [27–29]. Within 8–9 days after symptom onset, severe COVID-19 patients develop acute respiratory distress syndrome (ARDS) with aggressive inflammatory responses, hyaline formation, and pulmonary fibrosis [24, 30]. So, disease severity is mostly caused by the immune responses of the host immune system. In addition, the relation between growing severity and age is mostly alike to that of SARS-CoV and MERS-CoV [24, 26]. ARDS caused by SARS-CoV-2 is defined by the struggle in breathing, insufficient blood oxygen level, and also failure in the respiratory system that is responsible for death in 70% of lethal SARS-CoV-2 patients [31]. Some individuals may capitulate to secondary fungal and bacterial infections [26]. Furthermore, inflammatory cytokine storm caused by the immune system causes death (28%) followed by viral infection and/or secondary infections [31]. The majority of patients show mild symptoms; however, some patients develop ARDS likely triggered by a cytokine storm, increased inflammation, septic shock, failure in organs, and blood clots [32, 33]. Interaction between SARS-CoV-2 infection and immune cells leads to dysregulated immune responses and increased risk of multi-organ dysfunction. In SARS-CoV-2-infected individuals, different pro-inflammatory cytokines and other mediators like IL-2, IL-10, IL-7, IL-6, IL-1β, IFNγ, INF-ɑ, monocyte chemoattractant protein-1 (MCP-1), and induced protein 10 (IP10) significantly increased, causing failure in multi-organs [34–36] (Fig. 2). Increased level of cytokines and chemokines attracts several immune cells, especially T lymphocytes and monocytes, but not neutrophils, from the circulatory system into the infected tissue [37, 38]. The entry of immune cells into the lung tissue and the influx of lymphocytes into the airways may correlate with the high neutrophil-to-lymphocyte ratio, increased T helper-to-T regulatory cell ratio, and lymphopenia, which are present in approximately 80% of individuals [20, 39]. Furthermore, SARS-CoV-2 virus, which is a cytopathic virus, can induce injury and death in cells and tissues as a consequence of the viral life cycle [40]. Viral infection and replication in epithelial cells of the respiratory system may induce pyroptosis associated with vascular leak, as confirmed in people infected with SARS-CoV [41]. Pyroptosis, a caspase-1-based cell death, is an inflammatory type of programmed cell death that could be prompted by cytopathic viruses [42] and activates inflammatory response [43]. For example, IL-1β as a pyroptosis secretion is increased during SARS-CoV-2 infection [24]. Collectively, in addition to the lesion on pulmonary cells, the cytokine storm triggers inflammatory responses; thus, an increasing effort is focused on the regeneration of damaged cells as well as on the blocking or modulating of inflammatory responses.

## Extracellular vesicles

Extracellular vesicles (EVs) are heterogeneously bi-phospholipid encapsulated vesicles released from almost all eukaryotic cells, which mediate intercellular communication via transferring different biomaterial components like proteins, different kinds of nucleic acids, lipids, and carbohydrates between cells [44, 45]. Guidelines released by the International Society for Extracellular Vesicles (ISEV, https://www.isev.org) categorize EVs into three subtypes such as exosomes, microvesicles or shedding vesicles, and apoptotic bodies based on their origin and size. In recent years, scientists have a great attention to exosomes due to their pivotal roles in biological systems. Exosomes, 30–120 nm of EVs in size, are originating from endosome compartments (late endosomes) located inside the cytoplasm via a complex regularity system [46]. Once secreted, exosomes distribute into the extracellular matrix and bio-fluids and reach the neighboring and distantly located target cells [45]. Exosomes bear different molecules both on their surface and in the lumen. The density of sucrose (1.12–1.18 g/mL) is equivalent to that of exosomes, and they appear as cup-shaped vesicles under an electron microscopy [47]. As exosomes contain active biomolecules from parent cells, they can regulate function, fate, and shapes of target cells, participating in different pathological and physiological conditions [45, 48].

## Role of MSC-derived exosomes in SARS-CoV-2 treatment

MSCs, multipotent and self-renewal cells, are known as adult stem cells that can be obtained from different tissues such as the adipose tissue, bone marrow, umbilical cord tissue, and amniotic fluid. These cells are capable of differentiation into a variety of cells such as cartilage, bone cells, neural cells, muscle cells, and skin cells [49]. MSCs are widely considered to be the most useful tool in regenerative medicine because these cells can improve damaged tissues and organs via differentiation into different cells and also producing many types of chemokines, cytokines, growth factors, and EVs [50, 51]. MSCs may improve damaged lung tissue through homing toward specific injuries of the lung to maintain homeostasis as well as promote regeneration and secretion of soluble factors and exosomes to moderate inflammation and induce tissue regeneration [52, 53]. More recently, Leng et al. found that the transplantation of MSCs considerably improved the pulmonary function of patients with 2019-nCoV pneumonia over a period of 2 days [54]. They administered about one million cells per kilogram of weight once and declared that MSCs modulated immune responses, participating in the improvement of the outcome of SARS-CoV-2 pneumonia. Apart from transdifferentiation into tissue cells, the other mechanisms may involve in repairing tissue [55]; alternatively, paracrine effects (such as exosomes) of stem cells are the base of the beneficial effect of cell therapies [56].

**Fig. 3:**
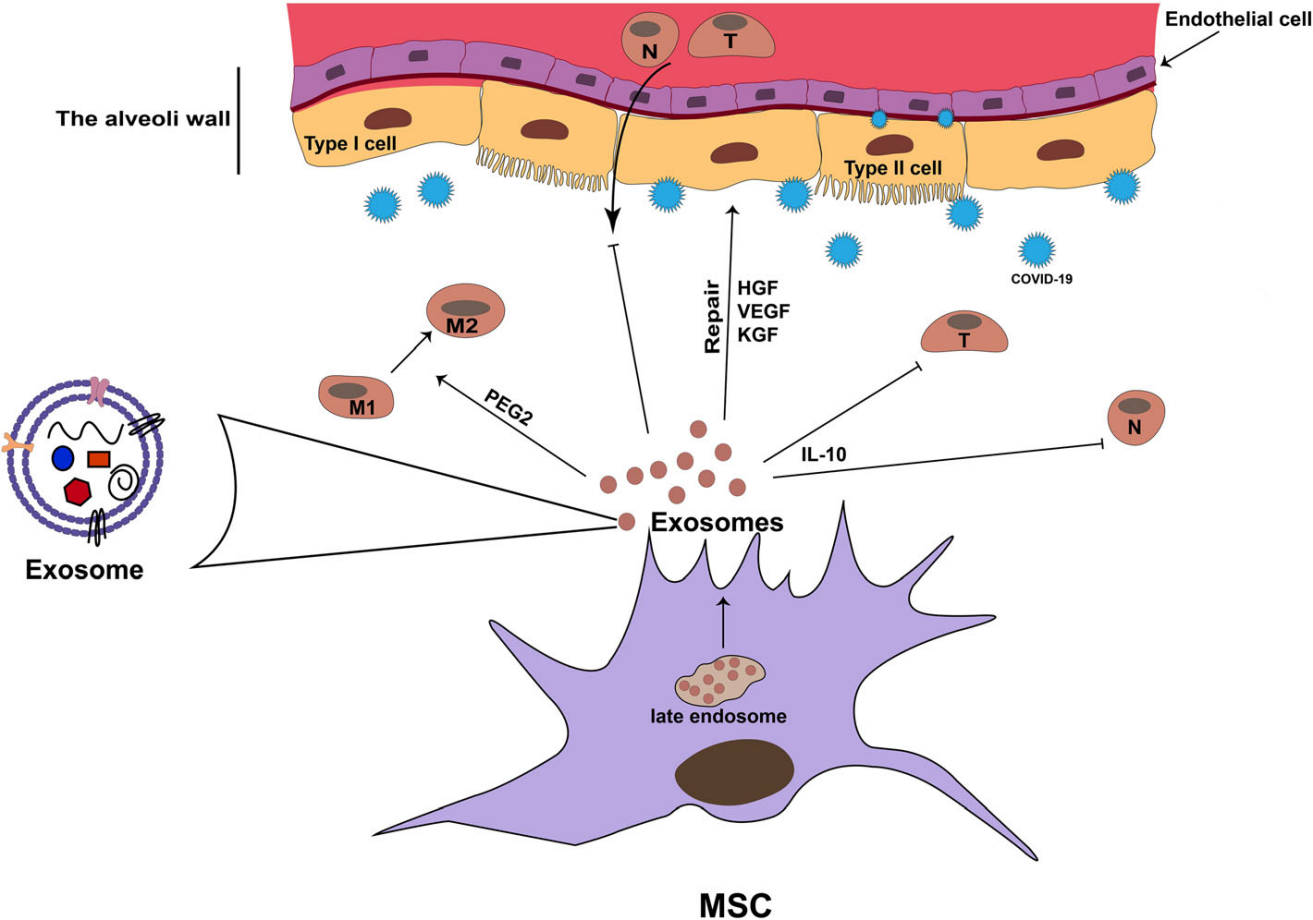
Possible mechanisms, by which mesenchymal stem cell-derived exosomes may improve adverse effects of SARS-CoV-2 virus infection. These exosomes contain different molecules that can suppress inflammatory responses and repair damage tissue. MSC, mesenchymal stem cell; N, neutrophil; M1, macrophage type I; M2, macrophage type II; T, T lymphocyte

According to the literatures, MSC-Exo contain different active biomolecules and can repair and improve myocardial infarcts, kidney injury, CNS diseases, liver cirrhosis, diabetic wound, and lung-associated diseases [57, 58]. The detailed mechanisms involved in exosome-mediated favorable effects and their site of action remain yet unclear. However, reported results from several pre-clinical non-SARS-CoV-2 models propose that MSC-Exo could also have efficiency against SARS-CoV-2 virus infection. For instance, through the systemic administration, MSC-Exo can modulate immune responses like increased cytokine storms in related acute lung injury and sepsis models [59–64]. Khatri and co-workers found that the administration of MSC-Exo attenuated influenza virus-induced acute lung injury through suppressing influenza virus replication in a pig model [65]. Similarly, a study conducted by Li and colleagues showed that MSC treatment significantly prevents avian H9N2-induced acute lung injury in mice via decreasing the level of chemokines and pro-inflammatory cytokines as well as reducing the infiltration of inflammatory cells onto the lungs [66]. In addition, in *Escherichia coli*-induced pneumonia mouse models, MSC-Exo had the ability to increase phagocytosis of bacteria [61, 67]. It seems that the hallmark of the treatment of SARS-CoV-2 severe pneumonia is to suppress the pro-inflammatory immune response induced by the virus, thus reducing the damage of both alveolar epithelial cells and capillary endothelial cells, followed by the restoring function of lung cells and also the recovery of the lung tissue which may be mediated by MSC-Exo (Fig. 3). Overall, as shown in Fig. 3, the possible therapeutic roles of MSC-Exo in SARS-CoV-2 virus infection may comprise inhibiting pro-inflammatory cytokines, inducing M2 macrophages through delivering PEG2, increasing secretion of anti-inflammatory cytokines like IL-10, and regenerating damaged tissue by producing KGF, VEGF, and HGF [6, 62, 68–71].

By June 2020, the public clinical trial database (https://clinicaltrials.gov) prepared three studies (NCT04276987, NCT0438938, and NCT04384445), and the Chinese Clinical Trial Registry site (ChiCTR, http://www.chictr.org.cn) records two clinical trials (ChiCTR2000030484 and ChiCTR2000030261) aiming to study the inhalation of aerosol and intravenous administration of MSC-Exo in the treatment of severe individuals with SARS-CoV-2 pneumonia. However, for SARS-CoV-2 pneumonia, further studies, which take MSC-Exo into account, will need to be undertaken, because various immune cells respond differently against MSC-Exo and these exosomes may modulate immune responses. For instance, Di Trapani et al. found that MSC-Exo strongly inhibited the function of B cells compared with the function of NK and T cells [72]. Therefore, the immunological activity of MSC-Exo toward different immune cells is dependent on the type of target cell, the cellular maturity, cellular status, and type of diseases among other factors [73, 74]. However, different studies declared that MSC-Exo can inhibit immune cell production and support an immunotolerant microenvironment.

## Other potential therapeutic applications of exosomes in 2019-nCoV treatment

This section provides some possible application of exosomes in SARS-CoV-2 virus infection treatment; however, there are some limitations regarding their application, which are discussed in the later section. In addition to exosome therapy, exosomes can serve as a drug delivery system for the treatment of SARS-CoV-2 virus pneumonia (Fig. 3). Exosomes exhibit superiority to other nanocarriers as they are bi-phospholipid vesicles, are of cell origin, safe, and have low immunogenicity and they can pass through the physiological barriers [75]. In this regard, different strategies can be used to construct optional exosomes such as (I) direct engineering method and (II) indirect engineering method [76].

**Fig. 4:**
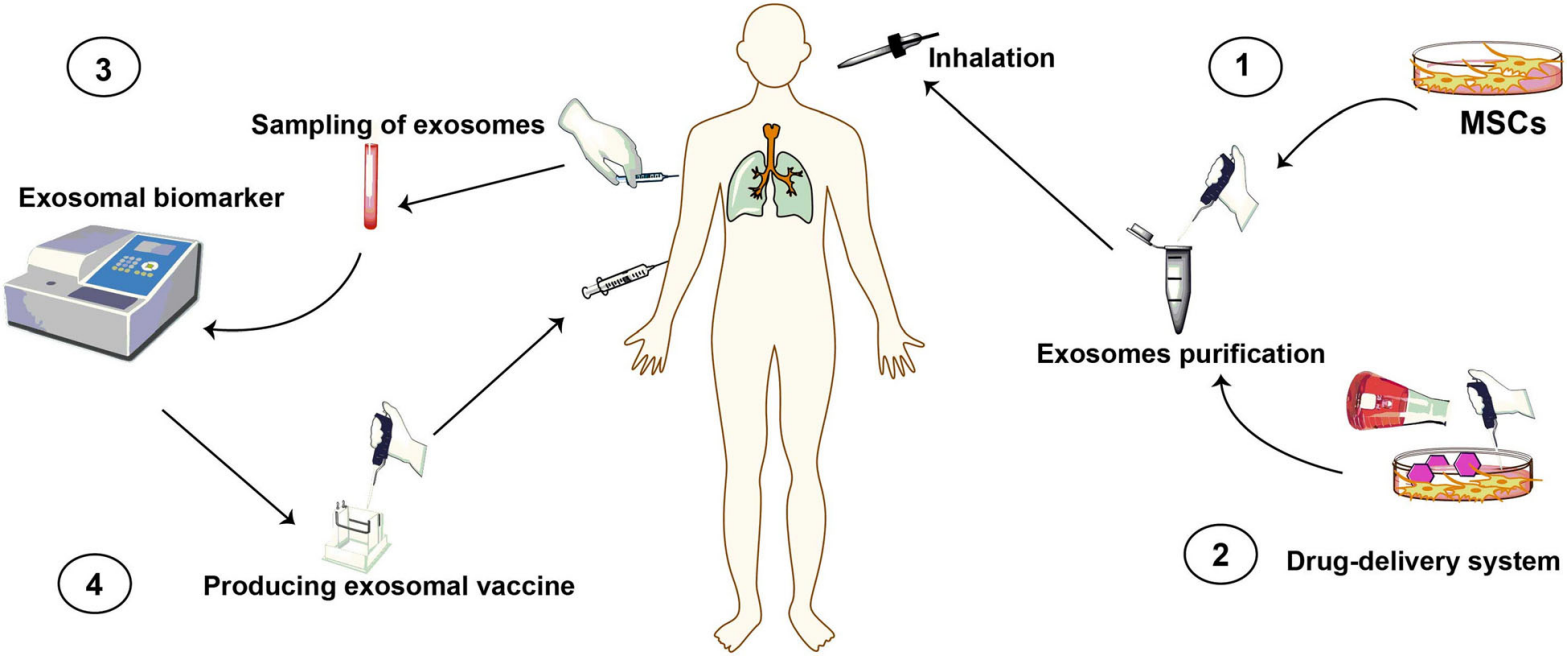
Therapeutic application of exosomes in the treatment of SARS-CoV-2 virus infection

By direct engineering methods, therapeutic agents (bio-molecules and drugs) are directly loaded into exosomes isolated from confident source cells, and then these exosomes are delivered into the tissue of target. By indirect engineering method, parent cells are co-cultured with therapeutic agents or genetically modified to produce artificial/drug-loaded exosomes. Pascucci et al., for instance, demonstrated the capacity of MSCs via microvesicles to package and deliver anti-cancer drugs such as paclitaxel, thus a potential tool for drug delivery. Interestingly, paclitaxel-loaded exosomes exerted a strong anticancer effect compared with exosomes isolated from the control cells [77]. Another possible application of exosomes is using them as a biomarker for SARS-CoV-2 virus infection. For other viruses, exosomes have been shown to transfer viral components such as proteins and miRNAs [78, 79]. Although the detailed mechanism by which SARS-CoV-2 viruses use exosomes for spreading and infection has not been explained exactly, it is reasonable that non-invasive sampling may be a new avenue to monitoring virus infection (Fig. 4). Furthermore, as mention above, exosomes from infected cells contain viral components that make them ideal for vaccine discovery. Although these exosomes could spread infection, a growing body of evidence indicates that the mentioned exosomes can induce immune responses [80]. It was demonstrated that exosomes derived from serum contain antigenic viral protein, suggesting the application of these exosomes as a novel vaccine approach [81]. If viral antigens are transferred on exosomes surface, it likely seems these exosomes serve as a vaccine for the treatment of SARS-CoV-2 virus infection (Fig. 4). Recently, exosomes have been shown to be inhibited both at the biogenesis and uptake level [82, 83]; therefore, inhibition of the biogenesis and secretion of exosomes from infected cells may reduce the speed and development of infection; however, upcoming studies are essential for confirmation. Collectively, in the case of MSC-Exo, the therapeutic application of MSC-Exo in COVID-19 disease may comprise using them as a drug delivery system as well as using them as therapeutic agents for suppressing inflammatory responses and regeneration of damaged tissues.

## Challenges

Exosomes have attracted many attentions by both academic and medical industry communities. The existence of different methods for EV isolation, purification, and characterization not only makes big challenges in the result analysis but also causes the conclusions to be unclear in the use of exosomes in clinical trials. Some of the previous studies did not use ISEV guidelines for the characterization of the EVs and assessment of their function, since those experiments have been accomplished prior to the 2014 and 2018 statements of ISEV guidelines of minimal experimental requirements for studies dealt with EVs [84, 85]. Although stem cell-based therapies represent some benefits such as availability and differentiation potential, they have several major hurdles for therapy [86], which may include (i) determining and targeting a reliable source of cells with phenotypic stability, (ii) the immunological incompatibility that may result in rejection, (iii) difficulty of handling and the higher cost of expansion, and (iv) the potential of cancer risks or formation of ectopic tissue. Exosome therapy exhibits several advantages against cell therapy such as the following: exosomes have a small size, which allows them to pass through filter organs including the liver, spleen, and lungs. Furthermore, as exosomes are bi-phospholipid vesicles, their membrane-binding property imparts excellent biocompatibility and biostability to the loaded cargos and optional drugs, and also, exosomes can pass through biological barriers like the brain-blood barrier. As a hopeful therapeutic tool for novel cell-free therapy, exosomes may serve as an alternative to stem cells in the treatment of various diseases for maintenance of the microenvironment for organ homeostasis and tissue regeneration upon injury [87, 88].

There are great developments in clinical exosome-based studies (https://clinicaltrials.gov); however, until now, no Food and Drug Administration (FDA)-approved exosome products are available (https://www.fda.gov/).

In the case of using MSC-Exo, from a clinical application point of view, a number of concerns should be considered before administering MSC-Exo to SARS-CoV-2-infected people. These comprise the source of MSC-Exo. MSCs, the heterogeneous cells, can be obtained from various tissues, even though MSCs isolated from the same sources may vary in entity and functionality [89, 90]. For example, several donor-derived bone marrow MSCs release exosomes with distinct cytokine profile content [91]. Besides, young MSC-Exo as compared with aged MSC-Exo improved LPS-induced acute lung injury in a mouse model [92]. In addition to immunomodulatory properties, MSC-Exo actually also regulate other biological processes, some with therapeutic functions [93], and others that may induce unexpected non-targeting effects. In order to inhibit the exosome biogenesis and secretion, future attempts should focus on silencing or eliminating exosomes that selectively encourage diseases which are not useful exosomes. Some research groups tried to use exosome inhibitors as versatile tools for exosome biology investigation; however, others have evaluated the inhibitory potential of such drugs in various disease models [82, 94]. Most of the experiments were carried out preclinical; therefore, clinical trials must be done to confirm their validity. However, the non-targeting effects of exosome inhibitors could be considered as the main issue on exosome biogenesis of healthy cells.

Another fascinating approach that exosomes can be used as a therapeutic agent is the drug delivery potential of them [95–97]. Exosomes from a safe source such as MSCs may be loaded with optional compounds or genetically engineered for targeting infected tissues/cells, suggesting the exosome-based nanocarriers for the treatment of infectious diseases. The advent of safe nanocarriers with high efficiency is the core goal of nanomedicine. However, most of the studies are designed in vitro and animal models; therefore, from a clinical trial application point of view, there are still more questions in the specificity, safety, and proficiency of this method. Moreover, despite various available delivery strategies for therapeutic anti-viral vaccines, therapeutic anti-viral vaccines in humans have been challenging to implement [98]. In another word, these vaccines have not been a strong success so far, and it is certainly valuable to consider more effective adjuvant methods for refining vaccine efficiency. Furthermore, another important thing is the source of immunostimulatory exosomes for human antiviral vaccines which needs more considerations [81]. In a nutshell, researches and explorations in the field of exosomes are in their early stages; therefore, to implement many of the aforementioned ideas, the biology of EVs (especially exosomes) from infected cells must be explored in-depth.

## Conclusion

Although the exploration of novel specific vaccine for the treatment of SARS-CoV-2 virus pneumonia is critical, it seems to take a long time. Therefore, further research into preventing and controlling the virus is essential as soon as possible. Exosomes from MSCs may be a useful tool for the treatment of SARS-CoV-2 virus pneumonia due to their pivotal roles in the suppression of inflammatory responses and the regeneration of damaged tissues. Furthermore, exosomes may serve as a biomarker, nanocarrier, and vaccine for the treatment of SARS-CoV-2 virus. However, for a better outcome, upcoming efforts are vital to carefully weigh the risk in the use of MSC-Exo for SARS-CoV-2 virus pneumonia against available pre-clinical findings in applicable in vivo models.

## Data Availability

The datasets used and/or analyzed during the current study are available from the corresponding author on reasonable request.

## Abbreviations

ARDS: Acute respiratory distress syndrome
EVs: Extracellular vesicle
FDA: Food and Drug Administration
IP10: Induced protein 10
MCP-1: Monocyte chemoattractant protein-1
MERS: Middle East respiratory syndrome
MSC-Exo: Mesenchymal stem cell-derived exosomes
SARS-CoV-2: Severe acute respiratory syndrome coronavirus 2
WHO: World Health Organization
